# Design and Simulation of On-Orbit Assembly System Based on Insect-Inspired Transportation

**DOI:** 10.3390/biomimetics8020256

**Published:** 2023-06-14

**Authors:** Yuetian Shi, Xuyan Hou, Guowei Gao, Zhonglai Na, Yuhui Liu, Zongquan Deng

**Affiliations:** Aerospace Manufacturing Engineering Department, Harbin Institute of Technology, Harbin 150001, China; qthshiyuetian@hit.edu.cn (Y.S.); 22s008182@stu.hit.edu.cn (G.G.); 22s108349@stu.hit.edu.cn (Z.N.); 22b908096@stu.hit.edu.cn (Y.L.); dengzq@hit.edu.cn (Z.D.)

**Keywords:** on-orbit assembly, dynamic modeling, space robot, flexible deformation

## Abstract

In response to the requirements of large-scale space in-orbit assembly and the special environment of low gravity in space, this paper proposes a small robot structure with the integration of assembly, connection, and vibration reduction functionalities. Each robot consists of a body and three composite mechanical arms-legs, which can dock and transfer assembly units with the transport spacecraft unit, and also crawl along the edge truss of the assembly unit to a designated location to complete in-orbit assembly while ensuring precision. A theoretical model of robot motion was established for simulation studies, and in the research process, the vibration of the assembly unit was studied, and preliminary adjustments were made to address the vibration issue. The results show that this structure is feasible for in-orbit assembly schemes and has good adjustment ability for flexible vibration.

## 1. Introduction

The research on space structures characterized by traits such as lightweight, high stability, and large scale has witnessed substantial growth owing to the rapid advancement of deep space exploration, crewed spaceflight, satellite communication, and other technologies among spacefaring nations worldwide. As a rising space power, many countries are actively participating in this field of research [1,2,3]. Modular in-orbit assembly of large structures is an effective method for resolving the launch mission of large structural bodies as the volume of transportation tools, such as rockets, is relatively small [4,5,6]. However, the current research on deployable structures cannot satisfy the development needs of future large or even ultra-large space structures in terms of mass, deployment aperture, and vibration characteristics. In-orbit assembly has become a research focus in the field of space technology in recent years [7,8].

Since the 1970s, major spacefaring nations have been researching the key technologies for the in-orbit assembly of large space structures, including the in-orbit assembly of the International Space Station and the use of modular units to construct large antenna systems for communication satellites [9,10,11,12,13,14]. Space science programs, Earth science programs, and human space exploration programs all require large space structures. The scale of space structures, such as large aperture space telescopes, kilometer-scale solar power platforms, and antennas with diameters exceeding 100 m, is huge [15,16]. In order to overcome the constraints posed by rocket launch vehicles and enable cost-effective and efficient in-orbit assembly of sizable space structures, it becomes imperative to employ foldable structures as supporting frameworks for the trusses of these expansive constructs. Consequently, a subsequent task involves executing the in-orbit assembly of modular units in a highly efficient manner within the confines of space [17,18,19].

However, the research on in-orbit assembly of large space structures is still in the exploratory stage both domestically and internationally. Although there are many studies on large deployable antennas, tension-based deployment mechanisms, and space-scale deployable mechanisms, the envelope diameter of current deployment mechanisms cannot meet the requirements of large-scale structure projects [20,21,22,23]. Therefore, it is crucial and necessary to conduct research on the design and simulation analysis of space deployable units for in-orbit assembly, which not only provides theoretical guidance for the in-orbit assembly of large space telescopes, solar power stations, and antennas, but also promotes the development of space technology and human progress [24,25,26].

NASA has made significant achievements in this field through its latest research, as evidenced by its successful experiment on satellite module self-assembly using space robots, demonstrating the potential of these robots to construct large structures [27]. The core module “Tianhe” of the Chinese space station is equipped with a 7-degrees-of-freedom redundant robotic arm, which consists of three wrist joints, three shoulder joints, and one elbow joint. This robotic arm is capable of autonomously assisting astronauts in performing in-orbit operations or maintenance tasks outside the capsule [28].

In-orbit assembly technology is a key research direction in future space exploration, but it presents challenges such as manufacturing large-scale structures, suppressing vibrations, and flexible assembly in space environments. To tackle these issues, multiple robot systems have been proposed to address the challenges of manufacturing large-scale structures [29,30]. Recent research has been focused on methods such as machine vision, bio-inspired algorithms, and reinforced learning, which have been investigated in assembly sequence planning, motion planning, and assembly methods. Additionally, there have been multi-robot collaborative motion planning algorithms for space robots and robot systems that differentiate between auxiliary and primary tasks. Nevertheless, a number of issues, including enhancing the intelligence of space robots, facilitating robot task allocation, and validating space robot systems on the ground, remain. Therefore, further research is required to strengthen the cooperative, perceptual, and interactive capabilities of space robots and to develop more accurate and viable ground validation methods to ensure successful in-orbit assembly [31,32].

The purpose of this paper is to propose an effective robot assembly plan that can achieve in-orbit assembly of super large components. The proposed assembly plan has significant theoretical and practical significance and will play an important role in future in-orbit assembly missions. In terms of research methods and ideas, we adopted a solution that combines legged robot mobility and mechanical arm gripping to complete three stages, namely, unloaded roaming, loaded roaming, and loaded assembly. We also established the dynamic model of the robot’s movement and assembly process and adjusted the impact of flexible elements on the assembled unit through dynamic simulation. The proposed robot assembly plan in this paper proves the feasibility and effectiveness of in-orbit assembly of super large components. Moreover, the adoption of robots for rail assembly can enhance efficiency, diminish expenses and hazards, and refine spacecraft design and structure. The established models and simulation findings are of immense significance for the development and enhancement of robot assembly design in the future.

## 2. Scheme Design of Robot System

**Figure 1:**
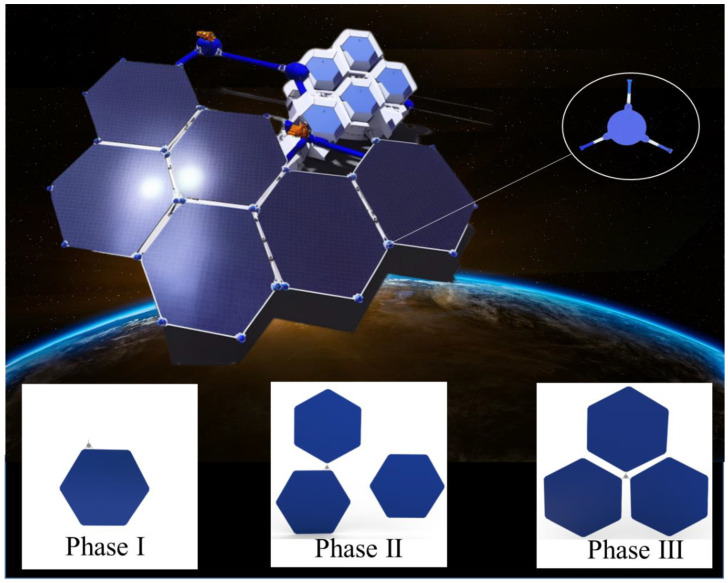
Schematic diagram of the conceptual on-orbit assembly scheme for space structure assembly units.

As shown in Figure 1, this paper presents a conceptual diagram of the in-orbit assembly of space structure assembly units. The hexagonal units, which are widely used in space structures, are selected as the assembly objects, and the robot structure with moving, handling, and assembly functions is used to connect the assembly units. Due to the special microgravity environment in space, the robot can make small adjustments and movements around the assembly unit. This new robot in-orbit assembly system can complete assembly tasks under autonomous control, enabling efficient, accurate assembly, connection, and transportation tasks, and is adaptable to different types of assembly units, with high flexibility and scalability.

The use of robots for in-orbit assembly can improve the efficiency and accuracy of assembly tasks compared to manual or traditional machine assembly methods. As shown in Figure 1, the three-stage workflow of in-orbit assembly robots is presented. During this process, the assembly robot assists in handling fixed connections and performs assembly work when the main robotic arm of the space station moves various assembly units to close proximity. The assembly robot can effortlessly handle large assembly units due to the unique weightless environment in space. The planning of robot assembly work is divided into three phases.

Phase One: The robot crawls along the edge of the unit truss to perform a cruising motion. At this time, the in-orbit assembly robot performs an unloaded cruising motion and moves laterally along the truss edge.

Phase Two: The robot grasps the assembled unit and performs cruising motion on the edge truss of another unit. At this time, the in-orbit assembly robot performs a loaded cruising motion, and the deformed swing of the grasped unit module due to its flexibility will affect the robot’s motion process. Thus, motion analysis is conducted, taking into account the effect of flexible units.

Phase Three: The robot moves to the preset pose to complete the assembly of the truss unit. At this time, on the basis of Stage Two, the in-orbit assembly robot holds one assembly unit per leg and uses the last leg to play the role of the manipulator to complete the in-orbit assembly of the assembly unit through posture adjustment.

In this paper, we propose a novel robot on-orbit assembly system for space structures to meet the task requirements of on-orbit assembly. The robot is composed of a body and three composite mechanical arm-legs, which can dock and transfer assembly units with the transport spacecraft unit. It can also crawl along the edge truss of the assembly unit to the specified position while ensuring accuracy for completing the on-orbit assembly.

**Figure 2:**
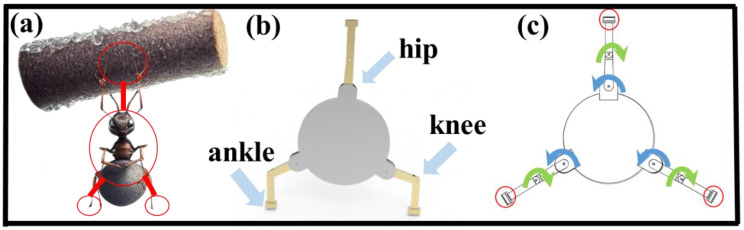
Structure of on-orbit assembly robot. (**a**) Schematic diagram of ants carrying heavy objects. (**b**) Configuration diagram. (**c**) Distribution of degrees of freedom.

The design of the robot is inspired by the ants in nature that carry heavy loads, as shown in Figure 2a. From a force perspective, when an ant stands upright, its body can support the weight of the load because its leg muscles can provide enough support. In addition, ants demonstrate flexible path-planning abilities during transportation. In space environments, path planning can be achieved through algorithms and intelligent control to avoid obstacles, maximize the use of space resources, and ensure efficient and safe transportation paths. Moreover, space environments present a variety of complex structures and special working spaces. By borrowing from the adaptive capabilities of ants, robots or mechanical devices can be designed with similar abilities to overcome challenges in space. In addition, ant transportation behavior also demonstrates efficient energy use and economy. Energy supply is a crucial issue in space environments, so in designing space transportation systems, optimizing energy use, reducing energy consumption, and extending system runtime can be taken into account.

This ant behavior is of great significance for robots engaged in space assembly, as they require similar mechanical stability and flexibility in order to manipulate and bear various different objects in zero-gravity environments. Robots in space can leverage the characteristics of zero-gravity and free-floating to perform assembly, maintenance, upgrades, and other tasks more efficiently, safely, and flexibly. Therefore, by drawing on the principles of ant-carrying behavior, new ideas and methods can be provided for the assembly and maintenance of robots in space.

The design of the robot was inspired by the way ants carry heavy objects in nature. Although ants are small in size, they can carry objects that are several times their own weight. Normally, ants use their front legs to grip objects and their hind legs to propel themselves backward. However, in narrow and elevated areas, they can lift and carry objects, as shown in Figure 2a. Based on this biological behavior, a robot system for on-orbit assembly was designed.

The overall structure of the system consists of the robot structure and the assembly unit. Considering that the assembly unit has a hexagonal structure with the hexagon vertices as nodes and three assembly units on each node, the in-orbit assembly robot adopts a tripedal configuration, with three legs symmetrically distributed around the center. Each leg is equipped with a gripping mechanism at the end. The configuration diagram is shown in Figure 2b. Since the three legs are symmetrically distributed around the center, any two legs can perform a patrol motion on the assembly unit.

The robot’s degree of freedom distribution is shown in Figure 2c. When the in-orbit assembly robot performs a patrol motion, its structure is similar to that of a bipedal robot, with the lower part of the robot consisting of two legs, including the thigh, calf, and foot.

## 3. Kinematics Model of Robot Moving Process

When studying the gait control of an on-orbit assembly robot, it is necessary to master the corresponding relationship between the angle of each joint and the trajectory of each joint, so it is necessary to establish the kinematics model of the robot. The robot kinematics model is the prerequisite for gait planning and control research.

**Figure 3:**
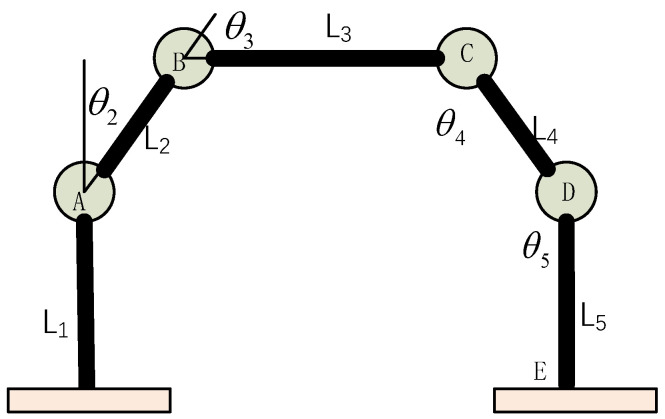
Five-link model of on-orbit assembly robot.

The patrol phase of the on-orbit robot can be simplified as a five-link model. Each leg includes the thigh, calf, and sole, and joints include the hip joint and knee joint. As shown in Figure 3, the links L1 and L2 constitute the left leg, the links L4 and L5 constitute the right leg, and the link L3 is the main body part of the robot.

The length of each connecting rod is set as *L_i_* (*i* = 1, 2, …, 5), the included angle between each connecting rod and the vertical direction is set as *I* (*i* = 1, 2, …, 5), wherein the clockwise included angle is set as positive, the center of the contact point between the left leg sole and the ground is taken as the origin to establish a coordinate system, the mass of each connecting rod is *m_i_* (*i* = 1, 2, …, 5), and the coordinate (*x_i_*, *z_i_*) (*i* = 1, 2, …, 5) is the center of gravity of the *i*th connecting rod.

Then, the coordinates at ankle joint A are:

(1) $$\left\{\begin{array}{l} x_{A}=0\\ z_{A}=l_{1} \end{array}\right.$$

Through the trigonometric function relationship, the coordinates of joints B, C, and D can be obtained.

(2) $$\left\{\begin{array}{l} x_{B}=x_{A}+l_{2}\sin\theta_{2}\\ z_{B}=z_{A}+l_{2}\cos\theta_{2} \end{array}\right.$$

(3) $$\left\{\begin{array}{l} x_{C}=x_{A}+l_{2}\sin\theta_{2}+l_{3}\sin\theta_{3}\\ z_{C}=z_{A}+l_{2}\cos\theta_{2}+l_{3}\cos\theta_{3} \end{array}\right.$$

(4) $$\left\{\begin{array}{l} x_{D}=x_{A}+l_{2}\sin\theta_{2}+l_{3}\sin\theta_{3}-l_{4}\sin\theta_{4}\\ z_{D}=z_{A}+l_{2}\cos\theta_{2}+l_{3}\cos\theta_{3}-l_{4}\cos\theta_{4} \end{array}\right.$$

The coordinates of each joint and the coordinates of the center of gravity of each connecting rod are derived with respect to time to obtain the speed of each point, and, finally, the coordinates of the center of gravity of the robot are obtained:

(5) $$\left\{\begin{array}{l} x_{g}=\frac{m_{1}x_{1}+m_{2}x_{2}+m_{3}x_{3}+m_{4}x_{4}+m_{5}x_{5}}{m_{1}+m_{2}+m_{3}+m_{4}+m_{5}}\\ z_{g}=\frac{m_{1}z_{1}+m_{2}z_{2}+m_{3}z_{3}+m_{4}z_{4}+m_{5}z_{5}}{m_{1}+m_{2}+m_{3}+m_{4}+m_{5}} \end{array}\right.$$

Inverse kinematics solution is a process of solving the rotation angle of each joint required to achieve this attitude by knowing the attitude coordinates of each point of the robot. According to the link length and joint position information, the solution formulas of the robot are deduced as follows.

(6) $$\theta_{2}=\arccos\frac{X_{B}^{2}+Z_{B}^{2}-L_{1}^{2}-L_{2}^{2}}{2L_{1}L_{2}}$$

(7) $$\begin{array}{l} \theta_{3}=\arccos\frac{X_{B}^{2}\;+\;Z_{B}^{2}\;-\;L_{1}^{2}\;-\;L_{2}^{2}}{2L_{1}L_{2}}\;+\\ \arccos\frac{{\left(Z_{C}\;-\;Z_{A}\right)}^{2}\;+\;{\left(X_{C}-X_{A}\right)}^{2}\;-\;L_{2}^{2}\;-\;L_{3}^{2}}{2L_{2}L_{3}} \end{array}$$

(8) $$\begin{array}{l} \theta_{4}=\arccos\frac{X_{B}^{2}\;+\;Z_{B}^{2}\;-\;L_{1}^{2}\;-\;L_{2}^{2}}{2L_{1}L_{2}}\;+\;\arccos\frac{{\left(Z_{C}\;-\;Z_{A}\right)}^{2}+{\left(X_{C}\;-\;X_{A}\right)}^{2}\;-\;L_{2}^{2}\;-\;L_{3}^{2}}{2L_{2}L_{3}}\\ +\;\arccos\frac{{\left(Z_{D}\;-\;Z_{B}\right)}^{2}\;+\;{\left(X_{D}\;-\;X_{B}\right)}^{2}\;-\;L_{3}^{2}\;-\;L_{4}^{2}}{2L_{3}L_{4}} \end{array}$$

(9) $$\theta_{5}=\arccos\frac{L_{4}^{2}+L_{5}^{2}-{\left(Z_{E}-Z_{C}\right)}^{2}-{\left(X_{E}-X_{C}\right)}^{2}}{2L_{4}L_{5}}$$

Therefore, through the above calculation, the forward and inverse kinematics equations of the five-link biped robot moving forward are obtained.

After establishing the kinematic connection between the robot’s components, it is necessary to plan the motion characteristics of each joint and the motion trajectory of the robot to ensure normal motion. Initially, the trajectory planning of the mobile components is conducted, with a focus on the moving legs during the single-leg support phase. The allowable rotation direction of each joint is predetermined to determine the robot’s trajectory, ensuring the stable movement of the robot.

To analyze the swing leg, firstly, the following four basic parameters are agreed upon: step size *S_L_*, single-leg support period time *T_S_*, the highest distance *H_m_* of the swing leg, and the corresponding horizontal distance *S_m_* when moving to the highest point. Other constraints used to design the trajectory of the swing leg are the repeatability of the gait and the impact of the leg when it falls.

Constraint 1: When the robot swings its leg to lift and fall, the end of the sole of the foot is located in the horizontal plane. The constraint equation can be expressed as:

(10) $$z_{a}(0)=0,z_{a}(T_{s})=0$$

Constraint 2: The maximum height of the robot raised by one leg cannot be too high *H_m_* to prevent possible collision. At this time, the vertical velocity is 0. The constraint equation can be expressed as:

(11) $$\begin{array}{l} x_{a}(T_{m})=S_{m}\\ z_{a}(T_{m})=H_{m}\\ {\dot{z}}_{a}(T_{m})=0 \end{array}$$

where *T_m_* is the time of movement when the leg is lifted to the highest point.

Constraint 3: The robot’s horizontal movement distance during a single-leg support phase is defined as the step size *S_L_*. To ensure a smooth transition between gait phases, the velocity of the robot’s position during the two-leg support phase and the one-leg support phase must be zero. Therefore, when switching from the two-leg support phase to the one-leg support phase, the initial velocity of the swing leg in the horizontal and vertical directions must be zero. This can be mathematically represented by the constraint equation:

(12) $$x_{a}(0)=-\frac{S_{L}}{2},x_{a}(T_{s})=\frac{S_{L}}{2}$$

(13) $${\dot{x}}_{a}(0)=0,{\dot{z}}_{a}(0)=0$$

Constraint 4: In order to reduce the impact of the swing leg landing, the horizontal and vertical speeds of the swing leg landing should be 0.

(14) $${\dot{x}}_{a}(T_{s})=0,{\dot{z}}_{a}(T_{s})=0$$

The three-order polynomial of the motion trajectory of the swing leg in the horizontal direction and the fifth-order polynomial in the vertical direction can be obtained by 11 simultaneous constraint equations satisfying the above four constraint conditions. The equations are as follows:

(15) $$\left\{\begin{array}{l} x_{a}(t)=a_{0}+a_{1}t+a_{2}t^{2}+a_{3}t^{3}\\ z_{a}(t)=b_{0}+b_{1}t+b_{2}t^{2}+b_{3}t^{3}+b_{4}t^{4}+b_{5}t^{5} \end{array}\right.\;\;\;\;\;\;0\leq t\leq T_{s}$$

## 4. Dynamic Analysis and Simulation of Patrol Process

In contrast to traditional biped robots, the on-orbit robot moves laterally on the solar panel and a complete walking cycle can be divided into two stages: the two-legged static support stage and the one-legged support stage. During the one-legged support period, one leg supports the other leg to move forward from the ground. The thigh rises first, followed by the lower leg falling. Then, the thigh falls forward and the lower leg swings backward until it touches the ground again.

Using the Lagrange method, the dynamic equation of the one-leg support period is established.

(16) $$D(\theta )\overset{..}{\theta }+H(\theta ,\dot{\theta })\dot{\theta }=T_{\tau }+T_{ext}$$

In the process of in-orbit assembly in space, the objects are analyzed in a weightless state, without any effect from gravity and with the disregard of potential energy. The symmetrical positive definite moment of inertia matrix is represented by $D(\theta )\in R^{5*5}$, while $H(\theta ,\dot{\theta })\in R^{5*5}$ stands for the matrix elements relating to centrifugal and Coriolis forces. The variables $\theta ,\dot{\theta },\overset{..}{\theta }\in R^{5*5}$ are generalized coordinates, velocities, and accelerations, whereas $T_{\tau }=[\tau_{1},\tau_{2},\tau_{3},\tau_{4},\tau_{5}]$ stands for the joint torque and $T_{ext}$ represents the externally applied torque. The expression of the matrix elements is as follows:

(17) $$\begin{array}{l} D_{ij}=p_{ij}\cos(\theta_{i}-\theta_{j})\\ H_{ij}=p_{ij}\sin(\theta_{i}-\theta_{j}){\dot{\theta }}_{j},i,j=1,2,...,5 \end{array}$$

Let $p_{ij}$ be defined as follows:

(18) $$p_{ij}=\left\{\begin{array}{ll} I_{i}+m_{i}d_{i}^{2}+a_{i}\left(\sum_{j=i+1}^{5}m_{j}\right)l_{i}^{2} & j=i\\ a_{i}m_{j}d_{j}+a_{i}a_{j}\left(\sum_{k=i+1}^{5}m_{k}\right)l_{i}l_{j} & j>i\\ p_{ji} & j<i \end{array}\right.$$

In the above formula, the mass, length, moment of inertia about the center of mass, and the distance from the center of mass to its rotating joint I of the *i*th link are, respectively, denoted by, and represented as *m_i_*, *l_i_*, *I_i_*, and *d_i_*. In addition, *a_j_* stands for

(19) $$a_{j}=\left\{\begin{array}{l} 0\;\;\;\;\;\;\;\;\;\;\;\;\;\;j=3\;\;\;\;\;\;\;\;\;\;\;\;\;\;\;\;\;\;\;\\ 1\;\;\;\;\;\;\;\;\;\;\;\;\;\;\;\;j=1,2,4,5\;\;\;\; \end{array}\right.$$

In the two-leg support period, the robot’s dynamic equation comprised a set of equations that constitute a Lagrange equation with a Lagrange multiplier and a closed chain constraint equation. This is due to the fact that the robot’s legs are positioned on the same horizontal plane.

(20) $$\left\{\begin{array}{l} D(\theta )\ddot{\theta }+H(\theta ,\dot{\theta })\dot{\theta }=J^{T}(\theta )\lambda +T_{\tau }\\ \Phi (\theta )=\left[\begin{array}{l} f_{1}\\ f_{2} \end{array}\right]=\left[\begin{array}{l} x_{e}-x_{b}-L\\ z_{e}-z_{b} \end{array}\right]=0 \end{array}\right.$$

The equation above can be described as follows: *J* is the Jacobian matrix, λ denotes the Lagrange multiplier, and *L* signifies the distance between the two feet during the two-leg support period, which is commonly known as the step size.

The stage between the single-leg support period and the double-leg support period is the impact stage. In this stage, the swinging leg touches the ground in an instant, and there will be an impact force between the sole of the foot and the ground, which will lead to a sudden change in the speed of the joint. Assuming that there is a plastic collision between the swing leg and the ground (there is no rebound when touching the ground), there is no relative sliding between the support leg and the ground. Then, the collision occurs in a very short moment, and the speed of the heel in the basic coordinates immediately becomes zero after the collision, while the position remains unchanged. Using the Lagrange shock model, there are:

(21) $$D(\theta )\ddot{\theta }+H(\theta ,\dot{\theta })\dot{\theta }=T_{\tau }+\delta F_{eut}$$

The equation defines $\delta F_{eut}$ as the generalized external force exerted on the leg during the swing phase when it makes contact with the ground.

In this paper, a cycle of one-leg movement for the robot is defined as follows: first, the thigh moves backward, followed by the folding of the lower leg, causing the foot to leave the ground to facilitate leg movement. The lowest point of the lower leg is maintained at a certain height from the ground. Then, the thigh starts to move forward, and the lower leg starts to extend and prepare to touch the ground. Next, the lowest point of the lower leg touches the ground to become a new contact point for the subsequent cycle. This cycle is repeated to complete the robot’s gait.

**Figure 4:**
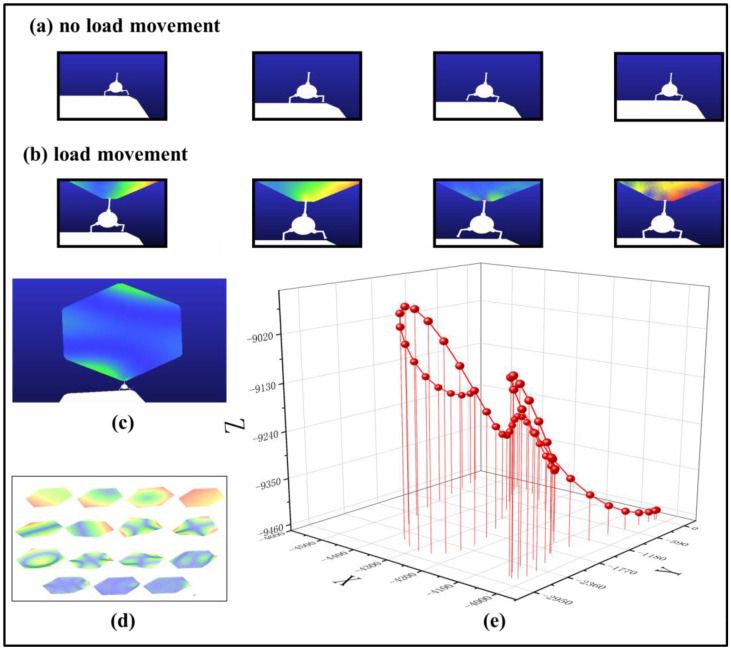
Simulation of the robot patrol process: (**a**) unloaded movement; (**b**) loaded movement; (**c**) vibration mode of the assembly unit’s flexible body under 15-order mode; (**d**) assembly unit with flexible deformation; and (**e**) oscillation amplitude of the assembly unit’s center of mass.

This process can be summarized into the following four stages, as shown in Figure 4a,b:

(1) Backward lifting of the thigh (L4): A gradually reduced clockwise torque is applied to the robot thigh, and the reduced speed (slope) is adjustable. As the torque exceeds the gravity received by the leg, the thigh begins to accelerate clockwise and the lower leg lifts off the ground while moving forward;
(2) Forward movement of the lower leg (L5): At the same time, a counterclockwise moment is applied. As the total moment decreases and becomes less than the moment generated by gravity, the leg moves counterclockwise and the lower leg continues to move forward and downward towards the thigh. Thus, the process of this movement can be converted into a change of torque;
(3) Forward falling of the thigh (L4): The thigh falls forward to adjust the posture and control the stability;
(4) Ground contact of the lower leg (L5): The lower leg relaxes to a certain extent and touches the ground, at which point the robot’s supporting leg is switched. As the inertial robot will lean forward, the lower leg touching the ground propels the robot forward for a certain distance. The other leg starts another cycle of one-leg movement, thus repeatedly realizing a cruising movement.

To validate the feasibility of the system design, a multibody dynamics simulation of the robot patrol process was conducted. The two-stage patrol process included unloaded and loaded patrols for the in-orbit assembly robot, and reasonable assumptions were made with simplified conditions. When the assembly unit is completed, it is considered a rigid body, while the unit to be assembled and held by the robot end-effector is treated as a flexible body. In order to investigate the influence of the flexible unit on the assembly process, a simulation analysis of the robot gripping and patrolling with the flexible unit was performed.

The paper employs the STEP function to control the driving of the robot in ADAMS. By implementing the STEP function, the continuity of velocity and displacement of the robot’s joint along the motion path is guaranteed. Maintaining trajectory planning, the study replaces the need for smoothing interpolation of motion parameters by configuring the joint’s rotation angles during specific time intervals. The simulation process lasts 18 s, with the robot’s legs lifted and lowered a total of eight times during two periods, requiring each joint to rotate at specific angles at different time intervals.

During the dynamic simulation of the loaded patrol movement in the robotic assembly, the assembly unit is dozens or even hundreds of times larger than the in-orbit assembly robot, so the assembly unit can cause oscillation during the robot patrol. Therefore, it is necessary to fully consider the flexible characteristics of the unit, and not analyze it as a rigid body. The simulation process is shown in Figure 4b.

This study examines a regular hexagonal assembly unit, which has been set as a flexible body and modal analysis carried out to discover the suitable modes for assembly. The unit was modally analyzed in various orders, and its 15-order vibration mode was shown in Figure 4c. Finally, the first six modes of the flexible body were determined and used as input loads in finite element analyses. Compared with rigid-body analysis, flexible-body analysis more accurately predicts the dynamic response of the assembly unit since its load significantly influences its output.

During the loaded patrol movement simulation, finite element analysis transformed the rigid body into a flexible body, and the robot’s grip caused a degree of oscillation, as shown in Figure 4d. By tracking the trajectory of the flexible body’s center points, as shown in Figure 4e, the degree of oscillation can be represented.

The data show that, after a period of measurement, the X-coordinate changed from −4556.158 to −4016.946, with a difference of 539.212 mm, and the Y-coordinate changed from −2784.092 to 22.0768, with a difference of 2806.169 mm. Meanwhile, the Z-coordinate changed from −9452.606 to −8943.167, with a difference of 509.439 mm. These changes are significant because they reflect the challenges the robot may face during the assembly task.

It is worth noting that the assembly units described in this paper are standard regular hexagonal units with a side length of 10,000 mm. The dimensions of the robot body are a radius of 500 mm. The center body of the robot comprises a rectangular prism and a cylinder with a radius of 100 mm, both connected to the thigh at a length of 150 mm. The thigh measures 425 mm in length, while the lower leg measures 350 mm. This implies that in the spatial realm of precise assembly tasks, the area of the assembly unit is more than 500 times larger than that of the assembly robot. Under special weightless conditions, only then can the system plan be potentially realized. It is therefore crucial to control flexible vibrations, as failure to do so will significantly impact the assembly accuracy and system stability.

To guarantee the assembly accuracy and stability of the system, effective control and stabilization measures must be taken for the robot, particularly in controlling flexible vibrations. With the implementation of appropriate control strategies and professional operational skills, errors can be significantly reduced, thereby ensuring the precision and stability of the robot during task execution.

## 5. Research on Robot Assembly Process

During the assembly process, the flexible assembly unit can exert continuously varying forces on the robot, causing the robot to undergo changes in force and displacement. To account for these changes, it is necessary to reconstruct the robot’s dynamics model.

The Lagrangian calculation method is used to analyze the work and energy involved in the robot system, solving the questions related to robot mechanics. The Lagrangian method analyzes only external constraint forces instead of internal constraint forces within the robot system. The method allows the establishment of coordinate systems according to the task.

The Lagrangian equation is expressed as follows:

(22) $$\frac{d}{dt}\left[\frac{\partial L}{\partial {\dot{\theta }}_{i}}\right]-\frac{\partial L}{\partial \theta_{i}}=Q_{i}\;i=1,2,3,\ldots ,m$$

where the meaning of *L* is the Lagrange function expressed by *V* and *T*, the physical meaning of *V* is kinetic energy, and the physical meaning of *T* is potential energy. *Q_i_* represents a generalized force, which does not contain potential. The method to determine this force is to find the virtual work done by the non-conservative force in the system, thus determining this force. *m* is the number of degrees of freedom of the system itself.

First, calculate the kinetic energy of the robot, take one of the moving legs as the research object, and establish a coordinate system {*L_i_*} in the *i*-th moving leg of the robot, then the speed of the *i*-th leg is:

(23) $$\begin{array}{l} V_{i}^{B}=J_{i}^{B}(\theta )\hat{\theta }\\ \begin{array}{l} T_{i}(\theta ,\dot{\theta })=\frac{1}{2}{\left(v_{i}^{B}\right)}^{T}N_{i}^{B}V_{i}^{B}\\ =\frac{1}{2}{\left(J_{i}^{B}(\theta )\hat{\theta }\right)}^{T}N_{i}^{B}J_{i}^{B}(\theta )\dot{\theta }\\ =\frac{1}{2}{\dot{\theta }}^{T}{\left(J_{i}^{B}(\theta )\right)}^{T}N_{i}^{B}J_{i}^{B}(\theta )\dot{\theta } \end{array} \end{array}$$

$N_{i}^{B}$ represents the inertia matrix of the *i*-th leg in the robot body coordinate system. $J_{i}^{B}$ represents the Jacobian matrix of the *i*-th leg in the robot body coordinate system. When discussing an inertial coordinate system, the description of the kinetic energy of the *i*-th moving leg is as follows:

(24) $$\begin{array}{l} T_{i}(\theta ,\dot{\theta })=\frac{1}{2}{\left(V_{i}^{S}\right)}^{T}N_{i}^{S}V_{i}^{S}\\ =\frac{1}{2}{\left(J_{i}^{S}(\theta )\dot{\theta }\right)}^{T}N_{i}^{S}J_{i}^{S}(\theta )\dot{\theta }\\ =\frac{1}{2}{\dot{\theta }}^{T}{\left(J_{i}^{S}(\theta )\right)}^{T}N_{i}^{S}J_{i}^{S}(\theta )\dot{\theta } \end{array}$$

$N_{i}^{S}$ represents the inertia matrix of the *i*-th leg in the inertial coordinate system. $J_{i}^{S}$ represents the Jacobian matrix of the *i*-th leg in the inertial coordinate system. Thus, it can be deduced that the total kinetic energy of the robot system is:

(25) $$T=\sum_{i=1}^{n}T_{i}(\theta ,\dot{\theta })=\frac{1}{2}{\dot{\theta }}^{T}N(\theta )\dot{\theta }$$

(26) $$\begin{array}{l} N(\theta )=\sum_{i=1}^{n}{\left(J_{i}^{B}(\theta )\right)}^{T}N_{i}^{B}J_{i}^{B}(\theta )\\ =\sum_{i=1}^{n}{\left(J_{i}^{s}(\theta )\right)}^{T}N_{i}^{S}J_{i}^{S}(\theta ) \end{array}$$

$N\left(\theta \right)$ represents the robot’s own inertia matrix. Under the constraint force generated by the assembly unit, the energy expression of the corresponding constraint force on the robot is:

(27) $$V(\theta )=\sum_{i=1}^{n}V_{i}(\theta )=\sum_{i=1}^{n}P_{i}{}^{T}r_{ci}(\theta )$$

In this Equation (27), *P* represents the constraint torque matrix. We obtain Equation (29) from Equation (28), which clarifies the relationship between the two equations.

(28) $$\begin{array}{l} \left[\begin{array}{c} {\bar{\omega }}_{i}^{}\\ \frac{\omega_{i}}{v_{ci}} \end{array}\right]=\left[\begin{array}{cc} I_{3} & 0\\ ad\left(r_{ci}\right) & I_{3} \end{array}\right]\left[\begin{array}{c} 0\\ \frac{\omega_{i}}{v_{ci}} \end{array}\right]\\ \;\;\left[\begin{array}{c} \vec{\omega_{i}}\\ \frac{\omega_{i}}{v_{ci}} \end{array}\right]=J_{i}\theta \end{array}$$

(29) $$\begin{array}{l} \frac{dV_{i}}{dt}={\dot{\theta }}^{T}J_{i}^{T}\left[\begin{array}{cc} I_{3} & ad^{T}\left(r_{ci}\right)\\ 0 & I_{3} \end{array}\right]\left(\begin{array}{c} 0\\ m_{i}g \end{array}\right)\\ ={\dot{\theta }}^{T}J_{i}^{T}\left(\begin{array}{c} P_{i}\times r_{ci}\\ P_{i} \end{array}\right)\\ \frac{dV_{i}}{dt}={\dot{\theta }}^{T}\frac{\partial V_{i}}{\partial \theta } \end{array}$$

$r_{ci}$ represents the position vector of the center of mass relative to the end effector. $ad\left(r_{ci}\right)$ denotes an operator that performs an affine transformation on vector $r_{ci}$. ${\bar{\omega }}_{i}^{}$ stands for angular velocity, $I_{3}$ stands for a 3 × 3 identity matrix.

Therefore, the Lagrangian function expression of the robot is:

(30) $$\begin{array}{l} L=T-V=\frac{1}{2}{\dot{\theta }}^{T}N(\theta )\dot{\theta }-V(\theta )\\ =\frac{1}{2}\sum_{i=1}^{n}\sum_{j=1}^{n}V_{i}(\theta )N_{ij}{\dot{\theta }}_{i}{\dot{\theta }}_{j}-{\sum_{=1}P_{i}}^{T}r_{ci}(\theta ) \end{array}$$

(31) $$\frac{\partial L}{\partial {\dot{\theta }}_{i}}=\sum_{j=1}N_{ij}{\dot{\theta }}_{j}$$

(32) $$\begin{array}{l} \frac{d}{dt}\left(\frac{\partial L}{\partial {\dot{\theta }}_{i}}\right)=\sum_{j=1}^{N}N_{ij}{\ddot{\theta }}_{i}+\sum_{j=1}^{dN_{ij}}{\dot{\theta }}_{j}\\ =\sum_{j=1}^{\infty }N_{ij}{\ddot{\theta }}_{j}+\sum_{j=1}\sum_{k=1}^{\ldots }\frac{\partial N_{ij}}{\partial \theta_{k}}{\dot{\theta }}_{k}{\dot{\theta }}_{j} \end{array}$$

(33) $$\begin{array}{l} \frac{\partial L}{\partial \theta_{i}}=\frac{1}{2}\frac{\partial }{\partial \theta_{i}}\left(\sum_{j=1}^{n}\sum_{k=1}^{n}N_{jk}{\dot{\theta }}_{j}{\dot{\theta }}_{k}\right)\\ +\sum_{j=1}^{n}J_{ij}^{T}\left[\begin{array}{c} P_{j}\times r_{cj}\\ P_{j} \end{array}\right]=\\ \frac{1}{2}\sum_{j=1}^{..}\sum_{k=1}^{..}\frac{\partial N_{jk}}{\partial {\dot{\theta }}_{i}}{\dot{\theta }}_{j}{\dot{\theta }}_{k}+\sum_{j=1}^{..}J_{ij}^{T}\left[\begin{array}{c} P_{j}\times r_{cj}\\ P_{j} \end{array}\right] \end{array}$$

From the above formulas, it can be concluded that:

(34) $$\begin{array}{l} \sum_{j=1}^{..}N_{ij}{\ddot{\theta }}_{j}+\sum_{j=1}^{..}\sum_{k=1}^{..}\frac{\partial N_{ij}}{\partial \theta_{k}}{\dot{\theta }}_{k}{\dot{\theta }}_{j}\\ -\frac{1}{2}\sum_{j=1}^{..}\sum_{k=1}^{..}\frac{\partial N_{jk}}{\partial {\dot{\theta }}_{i}}{\dot{\theta }}_{j}{\dot{\theta }}_{k}-\sum_{j=1}^{..}J_{ij}^{T}\left[\begin{array}{c} P_{j}\times r_{cj}\\ P_{j} \end{array}\right]=Q \end{array}$$

After simplification, it can be concluded that

(35) $$\begin{array}{l} \sum_{j=1}^{n}N_{ij}{\ddot{\theta }}_{j}+C_{ij}(\theta ,\dot{\theta }){\dot{\theta }}_{j}+P_{i}(\theta ,\dot{\theta })=Q_{i}\;\\ i=1,2,3,\ldots ,n \end{array}$$

Equation (35) is the Lagrangian equation for the robot’s dynamics. From left to right, the three terms on the left side of the equation represent inertial force, centrifugal force, Coriolis force, and the constraint force caused by the flexible body. The right side of the equation represents the Driven force.

**Figure 5:**
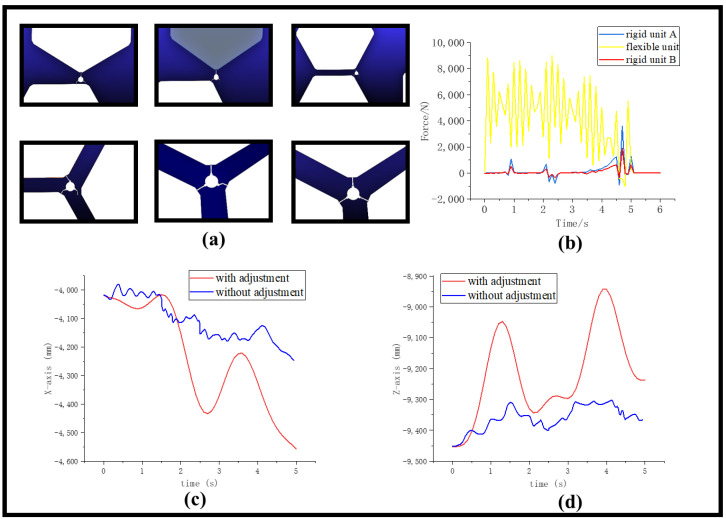
The robot assembly process and oscillation adjustment. (**a**) Six stages of the assembly process. (**b**) Change of the contact force between robot and flexible element. (**c**) Change of the central X-coordinate of the element after adjustment. (**d**) Change of the central Z-coordinate of the element after adjustment.

We have completed the simulation of the third phase of the on-orbit robotic assembly process using the previously described method. In the second phase, the robot grasps one unit and guides it in a cruising motion toward another unit. When it reaches the connection point of the two assembly units, one of the moving legs clamps down on the unit and adjusts its attitude to maintain a 120° angle with the load-bearing assembly unit. Meanwhile, the other moving leg functions as a robotic arm and grips the third assembly unit, thereby completing the on-orbit assembly process of the robotic system. Figure 5 illustrates a schematic diagram of the robotic system completing the assembly process.

The assembly process of the robotic assembly unit is demonstrated in Figure 5a. In the first stage, the robot moves the gripping device to the edge connector of the assembly unit and grasps the second piece of the assembly unit. In the second stage, the left-bottom gripping device of the robot moves to the connection point and secures it. In the third stage, the relative position of the two assembly units is adjusted to ensure that their edges are parallel to each other. In the fourth stage, the robot grasps the assembly unit and moves it to the location of the third assembly unit. In the fifth stage, the right bottom gripping device of the robot connects to the connection point of the third assembly unit. In the sixth stage, the robot adjusts its posture to meet the assembly requirements.

When a robot comes into contact with three assembly units simultaneously, it is necessary to measure the contact force between the robot and the units. During the simulation process, a comparison needs to be made of the force exerted at the connection between the rigid assembly unit and the robot, as well as the connection between the flexible assembly unit and the robot, as shown in Figure 5b. The force exerted on the identical locations of the flexible unit is more than 10 times that of the rigid unit and can reach up to 9000 N. This shows that the flexible vibration of the assembly unit can have a significant impact. During the simulation process, the gripping position is set in a stable state, but the actual operation must take into account the increased material requirements for the gripping device to withstand such a large force.

Uncontrolled deformations of the assembly unit can cause changes in its shape and position, resulting in an irregular surface that cannot be easily grasped by other components or robots. This can significantly increase the level of difficulty and the failure rate of the assembly process, adversely affecting the overall system accuracy. Therefore, a joint ADAMS and MATLAB simulation is employed to control the flexible deformation of the assembly unit. The central coordinates of the flexible unit are used as the output variable, while the input quantities are the angles of the hip and knee joints of the robot grip attached to the upper assembly unit. During the simulation test, the robot moves forward along the X-axis, while the grip points of the robot foot towards the Z-axis. The changes in the central unit in both the X- and Z-axis directions are detected, and the Z-axis changes in the central unit are adjusted through the angles of the hip joints in the feedback control loop, while the X-axis changes are adjusted through the angles of the knee joints. In this process, the two robot feet attached to the rigid unit are kept stationary, while the robot foot connected to the flexible unit is adjusted using the inverted pendulum principle to compensate for the flexible vibration of the assembly unit.

The specific control strategy is as follows: Define the desired changes in the central coordinates of the assembly unit, which correspond to the desired shape and position. During the simulation process, the actual changes in the central coordinates of the assembly unit are obtained by measuring the variations using sensors. The desired output is compared with the actual output to calculate the error, which represents the difference between the actual and desired outputs. Based on the magnitude and direction of the error, a control strategy is designed to adjust the joint angles of the robot. In the described scenario, the changes in the Z-axis direction can be compensated by adjusting the leg joint angles of the robot, while the changes in the X-axis direction can also be compensated by adjusting the leg joint angles. The desired output is compared with the actual output to calculate the error, which represents the difference between the actual and desired outputs. Using the control strategy, new joint angles are computed and applied as input to the system to adjust the shape and position of the assembly unit.

The X and Z coordinates of the center of the element were adjusted using the animation mode set to interactive, simulation set to continuous, and communication interval parameter for data exchange between MATLAB and Adams set to 0.05. The adjusted values are presented in Figure 5c,d.

The figures illustrate the substantial magnitude of the oscillations of the flexible element when left uncontrolled. These data can be considered an intuitive display of the effect of vibration on the flexible body. The uncontrolled vibration amplitude significantly increases, indicating that the flexible element is susceptible to disturbances that generate vibration signals. Moreover, the simulation data show that the shaking of the uncontrolled flexible element becomes more severe with time. However, by adjusting the robot control system, we can observe the damping effect of vibrations. This type of control can be viewed as an extension of traditional robotic technology that has great potential for both current and future assembly and manufacturing processes.

## 6. Conclusions

This paper proposes a robotic assembly solution for on-orbit assembly in space, featuring a lightweight and compact structure that enables the assembly of large components in the microgravity environment. The assembly process is composed of three stages: free-load cruising, load cruising, and load assembly, utilizing a quadrupedal robot’s movement and a manipulator arm’s grip to perform assembly tasks. Any two feet of the robot can be combined for bipedal movement, while a single foot can hold and assemble components. The study established the robot’s motion model and the dynamic model for the assembly process, completing dynamic simulations for all three stages. The simulations analyzed the influence of flexible elements on assembly units, and a simple adjustment was made for their oscillation. Results show that robots with a volume of 1% of the assembly unit can complete on-orbit assembly and effectively regulate the components’ flexible oscillation. However, the study has limitations, and the adoption of more advanced control algorithms could further improve the efficiency and stability of the assembly process. This research is of great significance to future on-orbit assembly tasks in space.

## Data Availability

The data that support the findings of this study are available from the corresponding authors upon reasonable request.
